# Burnout Syndrome Among Psychiatric Unit Nurses: Prevalence and Associated Correlates in Capital Territory Hospitals

**DOI:** 10.1155/jonm/4876394

**Published:** 2026-07-22

**Authors:** Arsalan Haider, Zahid Ullah, Li Hong

**Affiliations:** ^1^ School of Psychology, South China Normal University, Guangzhou, Guangdong, China, scnu.edu.cn; ^2^ The State Key Laboratory of Cognitive Science and Mental Health, Institute of Psychology, University of Chinese Academy of Sciences, Beijing, China, ucas.ac.cn; ^3^ Department of Psychology, University of Malakand, Dir(L) KPK, Chakdara, Pakistan, uom.edu.pk; ^4^ Nantong Vocational University, Nantong, jiangsu, China, ntvu.edu.cn

**Keywords:** burnout, capital territory hospital, comparative study, compassion fatigue, compassion satisfaction, psychiatry unit nurses

## Abstract

**Background:**

Burnout and compassion fatigue are crucial issues among the nursing workforce that are associated with compassion satisfaction and patient care behavior. Relatively little is known about burnout and compassion fatigue among psychiatric unit nurses (PNs) working in psychiatric units in developing countries, like Pakistan.

**Objective:**

This study examined and compared the prevalence of burnout, compassion fatigue, and compassion satisfaction among male PNs in public and nonpublic hospitals in Pakistan’s capital territory.

**Design:**

This study used a cross‐sectional research design.

**Methods:**

This study recruited male PNs from psychiatry inpatient units in public and nonpublic hospitals through purposive sampling between June 2023 and December 2023. The Professional Quality of Life (ProQOL) scale was used to assess PNs’ burnout, compassion fatigue, and compassion satisfaction levels.

**Results:**

Among PNs, 59% had severe burnout, 88% had higher risk compassion fatigue, and 12% had high compassion satisfaction. Correlation analysis revealed a significant negative association between burnout and compassion satisfaction (*r* = −0.261, *p* < 0.01). Intergroup comparison revealed that PNs in nonpublic hospitals demonstrated higher burnout (*M* = 28.46 vs. 26.98, *p* < 0.05, Cohen’s *d* = 0.330) and slightly higher compassion fatigue (*M* = 24.18 vs. 23.88, *p* > 0.05, Cohen’s *d* = 0.048). In contrast, PNs working in a public hospital reported higher compassion satisfaction (*M* = 32.53 vs. 29.62, *p* < 0.05, Cohen’s *d* = 0.442).

**Conclusion:**

The findings identify modest statistically significant cross‐sector differences in occupational well‐being indicators between PNs working in public and nonpublic hospitals, accompanied by small‐to‐moderate effect sizes. The tentative results highlight the necessity of targeted workplace interventions that may support the occupational well‐being of PNs operating within resource‐limited mental healthcare systems.

**Implications for Nurse Managers:**

This study underscores the necessity of targeted staff well‐being interventions within psychiatric inpatient units. PNs in nonpublic hospitals exhibited elevated burnout and diminished compassion satisfaction relative to their public hospital counterparts, whereas compassion fatigue affected nurses across both hospital types equally. Nurse managers can prioritize these disparities by implementing well‐being assessments, balanced workload distribution, and tailored psychological support programs. Boosting organizational support may mitigate burnout, enhance compassion satisfaction, improve staff retention, and promote high‐quality psychiatric care.

## 1. Introduction

Nursing is widely recognized as a highly stressful profession, often characterized by systemic workplace constraints, including limited resources, chronic understaffing, and elevated occupational demands. Excessive workloads and extended working shifts are pervasive challenges that correlate with increased stress, compassion fatigue (CF), and burnout symptoms among nursing professionals [1]. These global occupational stress challenges are disproportionately exacerbated within low‐ and middle‐income healthcare settings, particularly in Pakistan, which faces severe systemic nursing workforce shortages and resources.

Pakistan faces an acute national nursing workforce crisis. The country’s nurses‐to‐doctor ratio of 1–40 [1, 2], which substantially exceeds the World Health Organization’s (WHO) recommended ratio of 1:2 [3]. Pakistan maintains only 5.2 practicing nurses per 10,000 patients and produces fewer nursing graduates annually compared with neighboring South Asian countries, including Bangladesh and India [1, 2]. The national nursing deficit surpasses workforce shortages reported in several African nations [4]. Resultantly, work‐related stress and burnout are highly prevalent, with 30% of the Pakistani nursing workforce reporting severe workplace stress and 79% experiencing severe burnout [2, 5]. Long work hours, lower remuneration, and limited societal recognition further exacerbate occupational stress within the nursing profession than doctors receive [2, 6]. Persistent workforce shortages and high job demands have led to widespread chronic stress in frontline nursing staff.

In this context, stress is a psychological state characterized by irritability and emotional instability that arises when job demands exceed an individual’s abilities, thereby impairing physical, psychological, and occupational health [7, 8]. Persistent occupational stress is observable alongside burnout syndrome (BOS). Prolonged work hours, role ambiguity, and ongoing exposure to trauma are the key factors associated with nurses’ heightened vulnerability to burnout, particularly among nurses in nonpublic hospitals [5, 9]. Unlike many other healthcare professionals, who often engage in brief patient interactions, nurses provide continuous direct care and frequently witness suffering and death. This continual exposure to distressing situations is associated with emotional exhaustion and long‐term burnout if unaddressed [10]. While burnout and occupational distress are well‐documented among the nursing profession, evidence focusing specifically on registered psychiatric unit nurses (PNs) remains limited despite their unique and high‐risk job demands.

Despite the growing attention to burnout in healthcare, the unique role of PNs remains unexplored. Their responsibilities differ significantly from those of nurses in general medical units. For instance, PNs routinely encounter unexpected, uncertain, and unpredictable emotional and behavioral issues, and they face a higher risk of aggression and violent behavior from patients with psychiatric conditions, which ultimately increases burnout. Chronic, unpredictable workplace stressors heighten PNs’ risk of clinical burnout, a well‐documented multidimensional occupational psychological condition.

BOS is characterized by three interrelated domains. Maslach and Jackson [11] conceptualized BOS as a multifaceted condition involving (1) emotional exhaustion, or the feeling of being overextended and depleted of emotional energy; (2) depersonalization or the development of negative attitudes and responses toward others in the workplace; and (3) feelings of incompetence or a decline in feelings of personal and job performance that are associated with reduced personal achievement, that is, caregiving behavior [12, 13]. The nursing profession has comprehensive patient care responsibilities, including documentation and interprofessional collaboration. High workload and unsupportive work environments correlate with depleted psychological energy and elevated burnout symptoms [14].

Burnout symptoms manifest as chronic fatigue, headaches, sleep disturbances, irritability, and difficulty concentrating. These symptoms collectively impair productivity, increase clinical errors, and raise absenteeism [13, 15]. The key contributing factors include moral distress, an excessive workload, and a stressful environment, which are often intensified by high patient volumes and their behavior [16, 17]. These workplace stressors correlate with poorer nurses’ physical and mental health, reduce the quality of care, and diminish overall patient satisfaction, alongside higher CF prevalence [18].

CF is emotional and physical exhaustion resulting from sustained exposure to others’ suffering. It diminishes nurses’ capacity for empathy, motivation, and retention [19]. In contrast, compassion satisfaction (CS) is a state of positive feeling derived from helping others, performing their work effectively [20, 21]. Burnout and CF are negatively associated with CS, which is the sense of fulfillment derived from caregiving [20]. This compromises the quality of compassionate care, a core aspect of nursing practice [10, 22]. In the core aspect of care, inadequate coping mechanisms, role misalignment, and limited professional training are additional factors correlated with poorer occupational well‐being conditions [17, 23, 24]. To understand why burnout and CF disproportionately affect specific nursing subgroups, it is critical to examine the modifiable and demographic risk factors shaping occupational well‐being disparities.

### 1.1. Risk Factors and the Case of PNs

It is crucial to understand the risk profiles of individuals vulnerable to burnout to develop targeted interventions. According to empirical evidence, variables such as professional experience, area of specialization, type of work shift, and levels of job satisfaction are significantly associated with burnout risk [17, 25]. Sociodemographic characteristics, including gender, marital status, and socioeconomic background, also play a crucial role in increasing risk for burnout [10].

Job satisfaction refers to a positive subjective evaluation of one’s overall professional experiences and is influenced by workplace conditions, incentives, and institutional support [26, 27]. These organizational resources and policies differ substantially between public and nonpublic hospital systems, creating predictable gaps in nurses’ satisfaction between the two sectors. Furthermore, the socioemotional selectivity perspective suggests that older age and more experienced employees tend to exhibit higher resilience and more positive job attitudes than younger employees, who may struggle to adapt to demanding occupational conditions [10, 28–31].

Previous burnout studies have predominantly examined nurses from general medical units such as cardiology, surgery, and dermatology [10, 32]. However, occupational disparities in daily tasks and emotional demands are linked to varied burnout profiles in the nursing field. PNs, specifically, encounter distinct risk factors, including frequent contact with patients displaying unexpected and unpredictable behaviors and psychiatric symptoms [10, 33]. However, burnout‐related occupational stressors have been extensively studied in nursing specialties such as cardiology and surgery [10, 32]. Despite these unique vulnerabilities, minimal research has explored burnout, CF, and CS prevalence among male PNs, particularly within South Asian resource‐limited healthcare contexts.

This study aims to address BOS among PNs in public and nonpublic hospitals. Specifically, the study will determine the prevalence of BOS and investigate its association with CF and CS. Based on the literature review, the following hypotheses are proposed: *H1:* There is a positive association between burnout and CF, and a negative association exists between burnout and CS. *H2:* PNs working in public hospitals report higher CS levels than those working in nonpublic hospitals. *H3:* PNs in nonpublic hospitals exhibit higher levels of burnout and CF than those working in public hospitals, with longer work shifts and heavier workloads as potential correlational factors. Addressing these gaps in the literature, this study investigated occupational well‐being among male PNs and tested theory‐driven hypotheses regarding associations among relevant variables and workplace disparities.

This research is crucial because the international community currently lacks focused studies on this specific demographic. Further exploration is necessary to inform supportive policies and clinical practices.

## 2. Methods

### 2.1. Participants

This cross‐sectional study employed purposive sampling to gather the data. Prior to data collection, power analysis was performed using G ∗ Power 3.1 [34] to estimate an adequate sample size, with a medium effect size (*f* ^2^ = 0.15), *α* = 0.05, and power = 0.95.

### 2.2. Eligibility Criteria

#### 2.2.1. Inclusion Criteria

Participants were included in the study if they met all of the following criteria [1]: held valid registration with the Pakistan Nursing and Midwifery Council (PN&MC) [2]; possessed a nursing diploma or academic degree in nursing [3]; were 18 years of age or older [4]; had a minimum of 3 years of working experience within psychiatric units; and [5] were willing to provide both oral and written informed consent.

#### 2.2.2. Exclusion Criteria

Participants were not included if they met the following criteria [1]: fewer than 3 years of work experience in psychiatric units [2]; no active PN&MC registration [3]; refused to provide informed consent; or [4] were currently working at nonpsychiatric units.

### 2.3. Data Screening and Sample Attrition

A total of 110 males, registered as psychiatric nurses, were initially recruited from public and nonpublic hospitals located in the capital territory. Data collection took place from June to December 2023. Following data collection, standardized data quality screening was completed to remove invalid datasets. Ten participants were excluded from the initial sample for the following reasons [1]: failure to provide written informed consent (*n* = 2) [2]; withdrawal from the study before completion (*n* = 3); and [3] greater than 5% missing questionnaire responses (*n* = 5). After this data quality screening, the final analysis included 100 participants, with ages ranging from 20 to 52 years.

### 2.4. Ethical Approval

Ethical approval for this study was obtained from the Institutional Ethical Review Board (IRB) of the Capital Health Department and the Institute of Psychology at the University of Chinese Academy of Sciences, Beijing (approval no. 202304024). In accordance with the Declaration of Helsinki, all participants provided informed consent voluntarily, both orally and in writing.

### 2.5. Measurements

#### 2.5.1. Demographic Information

Trained clinical psychologists conducted semistructured interviews to collect sociodemographic characteristics, such as age, marital status, work experience, education level, socioeconomic status, and shift hours. This information was gathered on a demographics sheet.

#### 2.5.2. Job Satisfaction

Overall job satisfaction was assessed using a single‐item measure asking participants whether they were satisfied with their job. Responses were recorded as either “Yes” or “No,” with a “Yes” response indicating job satisfaction and a “No” response indicating job dissatisfaction.

#### 2.5.3. Professional Quality of Life (ProQOL)

The ProQOL‐5 scale was originally developed by Stamm [21]. The scale consists of 30 items with three domains [1]: CS (Items 3, 6, 12, 16, 18, 20, 22, 24, 27, and 30) [2], BOS (Items 1^∗^, 4^∗^, 8, 10, 15^∗^, 17^∗^, 19, 21, 26, and 29^∗^), and [3] secondary traumatic stress (STS: Items 2, 5, 7, 11, 13, 14, 23, 25, and 28). Each domain contains 10 items, and each item on the scale is answered on a 5‐point scale, ranging from 1 (*never*) to 5 (*very frequently*) over the last 30 days. Each domain composite score was obtained by summing all 10 items and the potential scores.

In the present study, the potential scoring was adopted to classify ProQOL indicators: burnout was classified into three levels: no BOS (≤ 18 points), moderate BOS (19–26 points), and severe BOS (≥ 27 points); CF was categorized into no fatigue and trauma risk (≤ 8 points), average risk (9–16 points), and high risk (≥ 17 points); CS was classified into low satisfaction (≤ 33 points), moderate satisfaction (34–41 points), and high satisfaction (≥ 42 points).

The ProQOL has good cross‐cultural applicability and stable psychometric features and has been widely culturally adapted and validated in Asian and South Asian healthcare populations. This measurement tool has been verified to have good reliability and construct validity among Chinese nursing interns [19], Pakistani healthcare professionals [35, 36], and local clinical psychologists [37], which supports its rationality for measuring occupational mental health status in the current research context. In the current sample, all three domains also yielded satisfactory internal consistency, with Cronbach’s alpha coefficients ranging from 0.73 to 0.80.

### 2.6. Statistical Procedures

Preliminary analyses were first conducted using SPSS Version 29. These analyses included data reorganization and cleaning, demographic analysis, and normality tests. Descriptive statistics were used to summarize categorical variables as frequencies and percentages, and continuous variables such as means with standard deviations. Based on the research hypotheses, we performed a Pearson correlation to assess the association among the study variables and an independent samples *t*‐test to assess group differences between public and nonpublic hospital PNs. Statistical significance was set at *p* < 0.05.

## 3. Results

### 3.1. Demographic Characteristics of the Participants

The results of Table 1 indicated that 60% of participants were between 20 and 35 years of age. Only 7% of the PNs had 16 years of higher education, and 42% of the participants belonged to lower SES. Sixty‐four percent reported extended working shifts up to 12 h. Sixty‐six percent of PNs in nonpublic hospitals showed signs of BOS. Forty‐six percent of PNs reported dissatisfaction with their work. Sixty‐one percent reported being single.

**TABLE 1:** Distribution of participants by age, education, employment setting, and work shift duration.

Variables	Percentages
Age (in years)	
20–35 years	60%
36–52 years	40%
Education attainment	
10 years	16%
12 years	43%
14 years	34%
16 years	7%
Socioeconomic status	
Low	42%
Middle	38%
High/rich	20%
Employment setting	
Nonpublic hospital	50%
Public hospital	50%
Work shift duration	
Standard shift (6–8 h)	36%
Extended shift (9–12 h)	64%
Burnout levels based on hospitals	
Nonpublic hospital	66%
Public hospital	34%
Job satisfaction	
Yes	46%
No	54%
Marital status	
Married	39%
Single	61%
Burnout levels	
Mild burnout	22%
Moderated burnout	19%
Severe burnout	59%
Compassion satisfaction	
No satisfaction	69%
Moderated satisfaction	19%
High satisfaction	12%
Compassion fatigue	
No risk for fatigue and trauma	5%
Average risk for fatigue and trauma	7%
High risk for fatigue and trauma	88%

*Note:* % = percentage.

The prevalence of burnout, CS, and STS was as follows: 59% had a higher level of severe burnout, only 12% exhibited CS, and 88% of PNs were at high risk for fatigue and trauma. Furthermore, nonpublic hospital PNs demonstrated a higher level of burnout (66%) compared to public hospitals.

### 3.2. Relationships of the Variables

Table 2 examined the association between the study variables among PNs. To assess normality, a prerequisite for conducting parametric statistical analyses, we examined skewness and kurtosis. According to Hair et al. [38], normality is deemed satisfied when the absolute value of skewness is less than 2, and the absolute value of kurtosis is less than 7. In the present study, all the study variables met the normality criteria, with skewness values ranging from −0.149 to 0.754 and kurtosis values ranging from −0.742 to 2.03; all values were well within the specified thresholds.

**TABLE 2:** Correlations among burnout, compassion fatigue, and compassion satisfaction among PNs.

Variables	Compassion fatigue	Compassion satisfaction	*α*	Scoring range		Skewness	Kurtosis
				Actual scores range	Potential scores range		
Burnout	0.497^∗∗^	−0.261^∗∗^	0.73	10–50	17–35	−0.149	−0.742
Compassion fatigue	1	−0.206^∗^	0.80	10–50	12–48	0.754	2.03
Compassion satisfaction	—	—	0.79	10–50	13–50	0.118	0.400

*Note:* Significant level ^∗^ *p* < 0.05, ^∗∗^ *p* < 0.01; compassion fatigue = compassion fatigue/secondary trauma.

Additionally, Pearson correlation analysis results revealed a positive association between BOS and CF (*r* = 0.497, *p* < 0.01). Conversely, BOS demonstrated a negative association with CS (*r* = −0.261, *p* < 0.01). CF was negatively associated with CS (*r* = −0.206, *p* < 0.05).

### 3.3. Comparison of the Hospitals

Table 3 shows that nonpublic hospital PNs reported a higher average burnout (28.46) than public hospital PNs (26.98). This cross‐sector difference reached statistical significance (*t* = 1.65, *p* = 0.041, Cohen’s *d* = 0.330), indicating a small‐to‐moderate effect size. This implies that less favorable workplace conditions in nonpublic hospitals are consistent with the job satisfaction supplementary question’s response. Conversely, public hospital PNs demonstrated a higher level of CS (32.53) than nonpublic hospital PNs (29.62), with a statistically significant difference (*t* = −2.20, *p* = 0.035, Cohen’s *d* = 0.442). The moderate small‐to‐medium effect size suggests that the difference in CS between the public and nonpublic hospitals is not only statistically significant but also meaningful in practical terms. This may support more supportive work environments and potentially reflect differences in organizational support, workload, or resource impact on nurses’ overall job and CS. There was no significant difference observed between the two groups of PNs in terms of CF (*t* = 0.239, Cohen’s *d* = 0.048). This implies that CF may be independent of hospital type in this sample, with negligible practical differences between the two groups.

**TABLE 3:** Differences in burnout, compassion fatigue, and compassion satisfaction across hospital types.

Variables	Nonpublic (*n* = 50)	Public (*n* = 50)	*t*	Cohen’s *d*
	M (SD)	M (SD)		
Burnout	28.46 (4.47)	26.98 (4.51)	1.65^∗^	0.330
Compassion fatigue	24.18 (6.40)	23.88 (6.14)	0.239	0.048
Compassion satisfaction	29.62 (6.71)	32.53 (6.46)	−2.20^∗^	0.442

*Note:* Significant level ^∗^ *p* < 0.05.

## 4. Discussion

### 4.1. Key Findings

The study examined the prevalence of BOS, CF, and CS among PNs. The study’s key findings are as follows: (1) higher educational attainment was associated with lower burnout risk; (2) more than half of the PNs reported longer work shift, burnout, being single, job dissatisfaction, and a higher risk for fatigue and trauma; (3) higher burnout rates were observed among PNs in nonpublic hospitals; and (4) PNs working in public hospitals demonstrated less CF and more CS. Additionally, effect size outcomes confirmed meaningful practical disparities in burnout and CS levels across study groups. These findings underscore the necessity of implementing tailored individual‐level risk mitigation strategies alongside institution‐based occupational well‐being interventions, including regular psychological debriefing sessions and structured resilience‐building training programs.

This study hypothesized that “*there is a positive* *association between burnout and compassion fatigue, and a negative association exists between burnout and compassion satisfaction*.*”* Our findings supported the first hypothesis: PNs who experience higher levels of burnout tend to lose their work energy more quickly, often becoming susceptible to CF. Prior studies align with our findings, documenting a negative correlation between severe CF and patient care behavior [39] as well as extended working hours [1]. Jeon et al. [40] likewise found that burnout correlates negatively with caregiving behavior. Burnout and CF are correlated with the compromised primary aim of nursing: patient care quality. Elevated burnout coincides with higher CF, and both correlate with poorer care performance and lowered CS among nurses. Zhang et al. [41], a meta‐analytic study, and the study by [20] both support the current hypothesis; they found a negative association between BOS and CS. Similarly, Hunsaker et al. [42] observed that insufficient staff support was negatively associated with higher levels of burnout and CF among American emergency department nurses. These studies indicated that multiple workplace factors were consistently correlated with burnout symptoms and CF among nursing professionals.

The second hypothesis of this study posited that “*Psychiatric unit nurses (PNs) working in public hospitals report higher compassion satisfaction levels than those working in non-public hospitals.*” The present findings supported our hypothesis, demonstrating that PNs in public hospitals reported lower burnout (34%, Tables 1 and 3) and higher CS (Table 3). In line with the calculated effect size, public hospital PNs exhibited slightly lower burnout severity relative to the nonpublic hospital counterparts, corresponding to small‐to‐moderate group differences. This pattern indicates that while burnout levels differ meaningfully across hospitals, the practical magnitude of these intersector disparities remains modest. The modest effect size suggests that hospital types explain a limited portion of the variation in burnout symptoms among male PNs.

The present findings align with prior evidence from Indonesia reported by Sudrajat et al. [5], which reported that PNs in nonpublic hospitals suffered more severe emotional exhaustion and depersonalization than those working in public hospital settings. Conversely, research conducted in Iran and Pakistan yielded contradictory findings, indicating that nursing staff in public hospitals face a greater risk of occupational stress and burnout [43, 44]. Similarly, a 5‐year longitudinal study documented elevated occupational stress among public hospital nurses, despite their reporting greater satisfaction [45]. Elevated CS among public hospital PNs is observed alongside better institutional facilities, team cooperation and support, and workplace incentives. In turn, it increases overall job satisfaction [27].

These inconsistent cross‐national findings highlight contextual discrepancies in occupational well‐being patterns. Unlike most previous studies that primarily focused on general nurses, the current research specifically evaluated burnout, CF, and CS among PNs. This professional subgroup is commonly burdened with diverse job roles, heavy workload responsibilities, and direct and indirect psychological stressors stemming from patient care and administrative tasks. These unique occupational traits correlate closely with adverse psychological mental health outcomes at the workplace [5, 9].

Consistent with present findings, Waqar and Hamid [46] confirmed higher job satisfaction among public hospital nurses in Pakistan, which was primarily associated with better facilities and stronger institutional workplace support. Furthermore, Nisa et al. [47] pointed out that while public hospital nursing staff exhibited lower burnout levels, they still endured persistent high occupational stress, highlighting the need for improved occupational support systems, and they consistently observed a high prevalence of stress‐related outcomes.

National workforce data confirm severe nursing shortages across Pakistan [3, 48]. However, epidemiological research focused especially on PNs remains scarce. The present sample represents 0.088% of Pakistan’s total registered nursing workforce, and findings are constrained by an exclusively single‐gender composition and regional recruiting scope. Accordingly, these findings apply exclusively to male PNs within the capital territory and cannot be generalized to female PNs or nursing staff in other regions. This sample provides valuable preliminary insights regarding occupational well‐being among this understudied subgroup, consistent with observations from recent regional studies [1, 2, 4].

The study’s third hypothesis posited that *“PNs in non-public hospitals exhibit higher levels of burnout and compassion fatigue than those working in public hospitals with longer work shifts and heavier workloads*.*”* Study results partially supported this hypothesis: nonpublic hospitals’ PNs reported moderately higher burnout and CF scores than their public hospital counterparts. However, the effect size for CF group differences was trivial, confirming that this cross‐sector disparity lacked both statistical significance and practical clinical meaning; only burnout yielded statistically significant, meaningful, and notable differences across public and nonpublic hospitals.

These findings are consistent with Sadati et al. [49], as well as those of others who observed burnout risk among public hospital nursing staff in Iran, and broadly suggest that workload pressure produces comparable burnout vulnerability between public and nonpublic hospital PNs. Contrary to the longitudinal work of Tyson and Pongruengphant [45], who claimed elevated burnout alongside greater CS for public hospital nurses, the current study found that public hospital PNs experienced lower burnout and higher CS overall. These findings may stem from institutional advantages unique to public care settings, including robust workplace support systems, modern clinical infrastructure, competitive remuneration, and formal staff incentives. Across nursing populations, burnout is commonly correlated with excessive workload, repeated exposure to patient suffering and mortality, and persistent job performance demands.

A local study in Pakistan’s capital corroborates the current pattern of elevated CS among public hospitals’ PNs, despite evidence that nonpublic hospitals’ facilities sometimes offer a flexible workplace environment paired with heavier patient care burdens. Waqar and Hamid [46] similarly linked public hospital nurses’ greater compensation satisfaction to flexible shift scheduling, higher monthly remuneration, expanded professional autonomy, and better hospital facilities, similar to the broader conclusions of [45]. Meta‐analytic and systematic review evidence further contextualized these occupational disparities: approximately 44% of obstetrics and gynecological nurses report lower personal accomplishment, influenced by sociodemographic characteristics, extended working hours, nurse‐to‐patient ratios, and frequent exposure to workplace verbal aggression [13, 50]. Collectively, the present study offers valuable context for clinical psychology and PNs, as it aims to address mental health burdens among PNs, whose roles involve chronic exposure to unpredictable, emotionally taxing workplace‐related stressors.

### 4.2. Limitations and Recommendations

Despite strengths, several limitations must be acknowledged. First, this study includes only male PNs. The findings cannot be generalized to female PNs, and all conclusions apply solely to male practitioners in the sampled region. Future studies should include both genders and participants from diverse geographical areas to enhance representativeness. Second, the current study had a relatively small sample of 100 PNs; future studies should collect larger and more diverse samples. Third, the study adopted a cross‐sectional design, which allows for correlational relationships among variables and precludes causal inferences. Future research should adopt mixed‐gender, multiregional sampling, implement larger sample sizes, and employ longitudinal prospective designs to explore causal pathways. Fourth, this study has employed a single self‐report measurement to measure burnout, CF, and satisfaction. Future studies should use larger sample sizes and more comprehensive tools to conduct relevant assessments. Finally, as this scale was developed within Western cultural contexts, it may carry inherent cultural biases. Variations in local occupational norms, working environments, and social values could influence respondents’ response patterns. Future research is recommended to develop a culturally adapted Urdu version and establish localized normative criteria to improve applicability in native populations.

### 4.3. Conclusion

This study identifies modest statistically significant cross‐sector differences in burnout and CF, associated with extended working shifts, heavy workload, and inadequate institutional recognition. These cross‐sector disparities are constrained by the small sample and modest effect sizes, so sector‐based conclusions should be interpreted cautiously. As correlational preliminary evidence, the findings offer tentative insights that may guide future exploratory policy reflections, refinements to staff incentive frameworks, and planning for targeted workplace mental health support measures. Greater institutional recognition for PNs may represent one tentative approach to support their staff’s occupational well‐being.

## Author Contributions

Arsalan Haider contributed to the study design, data curation, manuscript preparation, statistical analysis, and formatting. Zahid Ullah helped with data curation and data cleaning. Professor Li Hong was responsible for resources, supervision, reviewing the final draft, and approval.

## Funding

The study was funded by the Major Projects in Philosophy and Social Sciences of the Ministry of Education of China: 21JZD063.

## Disclosure

All authors reviewed and approved the final manuscript.

## Ethics Statement

The authors confirm that the study protocols were approved by the corresponding institute (approval no: 202304024) and adhere to the 2013 Helsinki Declaration.

## Consent

Written informed consent was obtained from all participants. As no minors were included in the study, parental consent was not required.

## Conflicts of Interest

The authors declare no conflicts of interest.

## Data Availability

All processed data used in this study can be obtained from the corresponding author upon reasonable request.
